# Intensity is not time: reframing dose prescription in post-stroke neurorehabilitation

**DOI:** 10.3389/fresc.2026.1796730

**Published:** 2026-05-29

**Authors:** Ibrahim Npochinto Moumeni

**Affiliations:** 1 Department of Physical Therapy & Physical Medicine, Faculty of Medicine and Pharmaceutical Sciences, University of Dschang, Dschang, West Region, Cameroon; 2 Department of Physical Medicine & Osteopathy, Regional Hospital of Bafoussam, Bafoussam, West Region, Cameroon; 3 Institute for Applied Neurosciences and Functional Rehabilitation (INAREF), Odza-Yaoundé, Cameroon; 4 Franco-African Center for Applied Rehabilitation and Health Sciences (CFARASS), Foumbot, West Region, Cameroon; 5 Department of Geriatrics and Gerontology, Sorbonne Université, Pitié-Salpêtrière Hospital, Paris, France; 6 Licensed Physiotherapy Practitioner, Paris, France; 7 Faculty of Health Sciences, University of Parakou, Parakou, Benin; 8 French-Speaking African Society for Neurorehabilitation (SAFNeR), Parakou, Benin; 9 UREKIM – Research Unit in Physiotherapy and Physical Medicine, Faculty of Medicine and Pharmaceutical Sciences, University of Dschang, Dschang, Cameroon; 10 CRESHDEM, Faculty of Medicine and Pharmaceutical Sciences, University of Dschang, Dschang, Cameroon

**Keywords:** constraint-induced movement therapy, dose-response relationship, motor recovery, neuroplasticity, neurorehabilitation protocols, repetition density, stroke rehabilitation, therapeutic intensity

## Abstract

**Background:**

Post-stroke neurorehabilitation remains predominantly prescribed according to session duration rather than biological effectiveness. However, converging evidence suggests that therapeutic intensity—defined as the density of neurofunctional stimulation per unit time—is the primary driver of neuroplastic adaptation and functional recovery.

**Objective:**

To synthesize contemporary evidence demonstrating the superiority of intensity-centered over duration-centered rehabilitation paradigms and to propose a pragmatic framework for clinical implementation across heterogeneous healthcare contexts.

**Methods:**

A structured synthesis of eleven contemporary studies (2010–2025) spanning randomized controlled trials, cohort studies, and integrative frameworks was conducted. Interventions modulating intensity through repetition density, neurophysiological load, temporal compression, and patient engagement were compared across high-income technological settings and low-resource family-mediated models.

**Results:**

Across all paradigms, interventions delivering higher repetition density, greater neurophysiological demand, and temporal compression consistently yielded superior functional outcomes compared with duration-matched controls. These effects were independent of technological sophistication or resource availability. An operational formula, *I* *=* *(R* *×* *L* *×* *E)/T*, is proposed to quantify therapeutic intensity, where *R* = task-oriented repetitions, *L* = neurophysiological load (motor, proprioceptive, metabolic, and cognitive demand), *E* = proportion of repetitions above the adaptive engagement threshold, and *T* = effective session time. A structured implementation model—the 3P Framework (Personalize, Progress, Prevent)—is proposed to guide dose calibration across patient profiles and recovery phases.

**Conclusion:**

Rehabilitation efficacy depends less on cumulative time than on the biological potency of stimulation delivered per unit time. The proposed 3P Framework—Personalize, Progress, Prevent—offers a pragmatic and evidence-based pathway to translate intensity science into routine clinical practice across patient phenotypes and care systems.

## What is known and what is new

### What is already known

- Neuroplasticity drives post-stroke motor recovery through cortical reorganization, synaptogenesis, and functional compensation, with peak responsiveness during subacute phases.
- High-repetition task-specific training improves outcomes compared to conventional therapy, as demonstrated across multiple randomized controlled trials in chronic stroke populations.
- Constraint-Induced Movement Therapy (CIMT) protocols deliver superior upper limb recovery when administered in compressed, high-intensity formats vs. distributed schedules.
- Duration-based rehabilitation prescriptions (e.g., “3 sessions per week for 12 weeks”) remain the dominant organizational model in clinical practice and reimbursement systems worldwide.
- Dose-response relationships in stroke rehabilitation are non-linear and depend on the biological potency of the intervention rather than its duration, as established through meta-analytic evidence across heterogeneous trial designs.
- Therapeutic nihilism among healthcare professionals—particularly in low- and middle-income countries—constitutes a documented barrier to rehabilitation uptake, independent of resource availability or patient motivation.

### What this study adds

- Operational definition of therapeutic intensity as a multidimensional construct integrating repetition density, neurophysiological load, active engagement, and temporal compression—moving beyond vague conceptual references.
- Phase-specific intensity thresholds (Table 2) translating neuroplasticity windows into actionable clinical targets: minimal effective intensity (*I*_min), target zones, and upper caution thresholds across acute, subacute, and chronic phases.
- Evidence that intensity is technology-independent: comparable outcomes can be achieved through robotic systems, conventional task-oriented therapy, or family-mediated models when repetition density and engagement are preserved.
- Resource-independence of intensity: high-density protocols implemented in low-resource African settings (2–4 h/day over 3–4 weeks) achieve functional gains (+43 Fugl-Meyer points) comparable to technology-intensive European protocols (+32 points over 12 weeks).
- The 3P Framework (Personalize, Progress, Prevent) as a structured implementation model operationalizing intensity prescription while integrating safety monitoring, fatigue management, and individual dose calibration.
- Systematic documentation that therapeutic pessimism, learned helplessness, and duration-centered delivery models constitute modifiable barriers to recovery—even in chronic stroke phases when intensity thresholds are exceeded.
- A quantifiable intensity formula—*I* = (*R* × *L* × *E*)/T—operationalizing therapeutic dose as a prescribable clinical variable, with explicit scoring criteria for neurophysiological load (L, four dimensions rated 1–3) and adaptive engagement (*E*, global session-level score 0–1), enabling reproducible dose comparison across sessions of identical duration.
- Empirical demonstration that the “Intensity Gap”—the systematic discrepancy between conventionally delivered intensity (*I* = 3–6) and the neuroplastic threshold (*I*_min)—is present across all post-stroke phases and represents a modifiable prescription failure rather than an irreducible resource constraint.
- First structured clinical example demonstrating the 80-fold difference in neuroplastic load between two sessions of identical duration (45 min)—Session A (*I* = 0.71, conventional) vs. Session B (*I* = 57.3, intensity-centered)—making the biological cost of duration-based prescription concretely visible for the first time.
- Reconceptualization of the rehabilitation professional's role from time provider to dose strategist: a paradigm shift with direct implications for curriculum reform, quality indicators, and reimbursement models in both high-income and resource-limited health systems.

## Introduction

Stroke neurorehabilitation remains paradoxically anchored to a time-based prescription model, despite decades of advances in neuroplasticity research (1–10). Across most healthcare systems, rehabilitation is still described, prescribed, and reimbursed in minutes and weeks rather than in biological dose. Typical prescriptions such as “30 min per session, three times per week” persist, not because they reflect neurophysiological evidence, but because they align with organizational convenience and administrative norms (8–14). Duration-based conventions emerged from institutional logistics rather than from neuroscience, and their perpetuation represents a structural barrier to optimal recovery (1–3, 15–20).

The biological basis for this critique is well established. Converging data across experimental, clinical, and translational studies demonstrate that time alone does not constitute therapeutic dose (1–3, 20–30). As Kwakkel et al. demonstrated in a seminal meta-analysis, the amount of therapy time explained only a modest proportion of variance in outcomes, whereas the quality and density of the stimulation delivered accounted for substantially greater gains (31). Lohse et al. further established through meta-regression that dose-response relationships in stroke rehabilitation are non-linear and depend on the biological potency of the intervention rather than its duration (32). Neuroplastic adaptation—whether expressed as synaptic strengthening, cortical reorganization, or functional compensation—depends primarily on the density and salience of neural activation, not on cumulative exposure time (1–13, 33).

Over the past fifteen years, multiple paradigms have independently challenged this duration-centered dogma. High-repetition task-oriented training, compressed constraint-induced movement therapy, high-intensity interval training, robotic massed practice, and more recently family-mediated intensive protocols all converge toward the same conclusion: intensity predicts recovery better than duration (1–11). Crucially, these effects are observed across phases of stroke recovery and across vastly different healthcare contexts, from highly resourced European centers to low-resource African settings (8–10).

Yet, despite this robust body of evidence, therapeutic intensity remains inconsistently defined, rarely quantified, and seldom prescribed explicitly. This conceptual ambiguity perpetuates systematic under-dosing and contributes to therapeutic stagnation, particularly in patients labeled as “chronic” or “plateaued” (10, 11, 29, 34). In practice, many so-called intensive programs are intensive only in duration, not in neurobiological demand. This failure of prescription—rather than failure of the patient—is the primary target of the present synthesis.

This Perspective argues that intensity should be considered the core unit of rehabilitation dose, reframes intensity as a multidimensional and quantifiable construct, and proposes a pragmatic implementation framework applicable across diverse clinical and socioeconomic contexts. Rather than advocating for longer therapy, it calls for smarter, denser, and biologically targeted rehabilitation.

## Evidence synthesis

### From duration-centered to intensity-centered neurorehabilitation paradigms

#### Why duration fails as a surrogate for therapeutic dose

Across post-stroke rehabilitation literature, session duration has long been used as a proxy for therapeutic dose. However, duration alone fails to capture the neurobiological requirements necessary to trigger adaptive plasticity. Two interventions of identical length may differ radically in their capacity to activate motor networks, recruit attentional resources, and induce synaptic change. As a result, duration-based prescriptions systematically underestimate the true variability of rehabilitation dose delivered in clinical practice (1–25, 31, 32).

Several landmark studies demonstrate that when duration is held constant, manipulations of intensity alone are sufficient to generate markedly different outcomes. This observation holds across acute, subacute, and chronic phases of stroke recovery and challenges the assumption that longer therapy necessarily equates to more effective therapy.

### Converging evidence from contemporary rehabilitation paradigms

#### High-repetition and task-oriented training

Experimental and clinical work on task-specific upper limb rehabilitation consistently shows that repetition density is a primary determinant of recovery. Studies contrasting conventional therapy (typically 30–50 repetitions per session) with high-repetition paradigms (300–600 repetitions per session) report substantially greater improvements in motor impairment and functional use, despite comparable session durations (3, 5). These findings highlight that neural systems respond to repetition thresholds rather than elapsed time. Recent evidence from technology-assisted high-repetition paradigms further confirms that the number of meaningful task attempts—not the technological sophistication of the delivery platform—drives cortical reorganization (34, 35).

#### Temporal compression and distributed practice

Constraint-induced movement therapy provides a particularly illustrative model. When the same therapeutic volume is delivered through temporally compressed protocols (several hours per day over a short period), outcomes are superior to those achieved with distributed, low-density schedules spread over weeks (4). This suggests that temporal compression amplifies neuroplastic signaling, likely by maintaining sustained network activation within critical windows of motor relearning.

#### Physiological load and cardiovascular intensity

Beyond motor repetition, cardiovascular and metabolic load further modulate neuroplastic responsiveness. High-intensity interval training protocols demonstrate that increasing physiological demand—while keeping session duration constant—leads to superior gains in aerobic capacity, gait performance, and functional endurance (2, 7). Importantly, these effects are observed even in the acute phase of stroke when appropriately monitored, refuting longstanding concerns that high intensity is intrinsically unsafe early after stroke (7).

#### Technology-driven density vs. time exposure

Robotic-assisted therapy and high-frequency telerehabilitation illustrate another critical point: technological sophistication does not enhance outcomes through time extension but through increased practice density and consistency. Large multicenter trials show that robotic or home-based digital interventions achieve benefits comparable to or exceeding conventional therapy primarily because they enable sustained, high-density task execution rather than longer sessions (5, 6, 36).

#### Transcultural validation of the intensity principle

Perhaps most compelling is the observation that intensity-driven effects are robust across resource settings. Comparative Franco–Cameroonian cohorts and family-mediated intensive protocols demonstrate that when intensity is preserved—through repetition density, proprioceptive load, and active engagement—functional outcomes rival those achieved in high-income, technology-rich environments (8–10). These findings strongly support the notion that intensity is a biological principle rather than a technological privilege.

Family-mediated models further reveal that intensity can be multiplied without proportional increases in professional staffing by shifting selected therapeutic tasks to trained caregivers, thereby extending effective stimulation while maintaining quality and safety (9). Critically, this transcultural validation challenges the false premise that advanced rehabilitation is exclusively the province of well-resourced settings. The Cogni-Famille protocol, delivering over five hours of daily stimulation through trained family members, achieved Fugl-Meyer gains of +43 points compared to +32 points in technology-rich European centers (9). This convergence across radically different contexts constitutes among the most powerful evidence that intensity—not technology—is the active ingredient in neuroplastic recovery, see Table 1.

**Table 1:** Intensity-Centered vs. Duration-Centered Paradigms in Post-Stroke Rehabilitation.

Study	Design	Phase	Intervention	Intensity manipulation	Key outcomes	Core message
Ward et al. (1)	Prospective cohort (*n* = 224)	Chronic	90 h/3 weeks task-specific training	Temporal compression, high repetition density	ARAT, WMFT, FMA-UE	Intensity predicts recovery better than total hours
Boyne et al. (2)	RCT (*n* = 40)	Subacute	HIIT vs moderate cycling	Cardiovascular load ↑, same duration	VO₂peak, gait speed	Physiological intensity drives adaptation
Waddell and Lang (3)	Repeated cohorts	Chronic	300–600 vs 30–50 reps/session	Repetition density ↑	FMA-UE, MAL	Repetition number is a key determinant
Corbetta et al. (4)	Cochrane review	Mixed	CIMT compressed vs distributed	Same volume, compressed delivery	WMFT, FMA-UE	Temporal compression enhances efficacy
Lo et al. (5)	Multicenter RCT (*n* > 350)	Subacute	Robotic massed practice	High density despite short sessions	FMA-UE, ADL	Density outweighs session duration
Cramer et al. (6)	RCT (n≈700)	Subacute	High-frequency telerehab	Frequency and engagement ↑	ARAT, grasp	High-density home training is effective
Amanzonwé et al. (7)	RCT (*n* = 165)	Acute	HIIT + PT vs simulated cycling	Intensity ↑, duration controlled	mRS, 6MWT	High intensity is safe and superior
Moumeni (8)	Comparative cohort (*n* = 78)	Acute→Chronic	2–4 h/day high-density therapy	Intensity amplified, limited duration	FMA-UE, BI, gait	Rapid gains despite limited resources
Moumeni et al. (9)	Retrospective (*n* = 62)	Subacute/Chronic	Cogni-Famille protocol	Family multiplies intensity >5 h/day	FMA-UE, FIM	Outcomes comparable to high-tech centers
Moumeni (10)	Integrative synthesis	Mixed	Multimodal intensive framework	Density + compression + engagement	Motor and functional scales	Synchronizing thresholds maximizes recovery
Moumeni (11)	Integrative perspective	All phases	Intensity-over-duration paradigm	Unified: density, load, compression, engagement	Functional recovery, dose-response	Intensity unifies heterogeneous recovery paradigms

Eleven studies spanning randomized controlled trials, cohort studies, and integrative frameworks. Across all paradigms, therapeutic intensity—operationalized through repetition density, physiological load, engagement, and temporal compression—consistently outperforms duration-based prescriptions. RCT, randomized controlled trial; FMA-UE, Fugl-Meyer Assessment Upper Extremity; ARAT, Action Research Arm Test; WMFT, Wolf Motor Function Test; MAL, Motor Activity Log; BI = Barthel Index; FIM, Functional Independence Measure; HIIT, high-intensity interval training; 6MWT = six-minute walk test; mRS, modified Rankin Scale.

#### Interim synthesis

Taken together, these converging lines of evidence undermine the validity of duration as the primary unit of rehabilitation dose. Whether intensity is achieved through repetition density, cardiovascular load, temporal compression, technological facilitation, or family-mediated amplification, the determinant of recovery remains the same: the amount of neuroplastic load delivered per unit time. This convergence sets the stage for a necessary conceptual shift—from describing rehabilitation in minutes and weeks to prescribing it in biologically meaningful units of intensity.

## Operationalizing therapeutic intensity

### From an abstract concept to a prescribable clinical dose

While the evidence synthesized above consistently demonstrates the superiority of intensity-centered over duration-centered paradigms, a major barrier to clinical translation remains: intensity is rarely operationalized. In routine practice, clinicians are encouraged to “increase intensity” without clear guidance on how to define, quantify, or monitor it. As a result, intensity remains an intuitive notion rather than a prescribable therapeutic parameter.

To bridge this gap, intensity must be reframed as a multidimensional construct, integrating the key determinants repeatedly identified across the studies summarized in Table 1. These determinants converge around four core dimensions: repetition density, neurophysiological load, active engagement, and time.

### A pragmatic quantification model

Based on the converging evidence and clinical experience synthesized in the Franco–Cameroonian and family-mediated models (8–11), therapeutic intensity can be operationalized using the following formulation:

I=(R×L×E)/T

**Where:** *R* = number of task-oriented repetitions performed during the session; *L* = neurophysiological load across four independent dimensions—motor, proprioceptive, metabolic, and cognitive—each scored on a 3-point scale: 1 = low demand (simple repetitive unresisted movement, minimal attentional requirement); 2 = moderate demand (task-specific movement against gravity or with resistance, requiring sustained attention); 3 = high demand (complex multi-joint coordination, dual-task or cognitively loaded exercise, near-maximum effort). The four scores are summed to yield the composite L (range 4–12); E = proportion of repetitions performed above the adaptive engagement threshold (range 0–1), where E = 0 indicates that no repetition reached threshold—typically when the patient is passive, disengaged, or performing below the minimum cognitivo-motor demand required to activate neuroplastic cascades—and *E* = 1 indicates all repetitions were at or above threshold. *E* is a global session-level score, not assessed separately per dimension of L, and is estimated from three observable indicators: task success rate (proportion of attempts meeting predefined accuracy criteria), fatigue monitoring (absence of qualitative decline in movement quality across the session), and patient responsiveness (active participation without prompting)*T* = effective session time in minutes, excluding passive transitions and resting intervals.

This formulation does not aim to provide a rigid mathematical score, but rather a clinical reasoning framework that allows therapists to compare sessions of identical duration but vastly different biological potency. Two sessions lasting 45 min may differ by an order of magnitude in delivered neuroplastic load, depending on how these parameters are combined, see Table 2.

**Table 2:** Clinical example (added in revision). Both sessions last 45 min. Session A reaches an intensity score of 0.71—well below the minimal effective threshold for the subacute phase. Session B, restructured around task-specific practice and engagement, achieves a score of 57.3—within the target zone for subacute neurorehabilitation. This 80-fold difference in neuroplastic load would be invisible under duration-based prescription. L is rated on a composite scale: motor (1–3) + proprioceptive (1–3) + metabolic (1–3) + cognitive (1–3); *E* is estimated from task success rate and fatigue monitoring. These values are illustrative; validation of the scoring system in prospective studies is warranted.

Session A—Conventional (Duration-Centered)	Session B—Intensity-Centered (Same Duration)
Duration: 45 min|*R* = 40|*L* = 2|*E* = 0.4|*T* = 45 *I* = (40 × 2 × 0.4)/45 = 0.71 *Mostly passive mobilization, frequent rest, low task specificity. Below minimal neuroplastic threshold.*	Duration: 45 min|*R* = 350|*L* = 8|*E* = 0.78|*T* = 38 (active) *I* = (350 × 8 × 0.78)/38 = 57.3 *Task-specific reaching, proprioceptive load, verbal engagement, minimal passive intervals. Within target intensity zone.*

### Clinical application example

#### Clinical implications of intensity quantification

The implications of this model are substantial. First, it explains why prolonged low-density sessions often fail to induce meaningful recovery, particularly in chronic stroke. When repetition thresholds and engagement levels remain below adaptive levels, increasing duration alone does not compensate for insufficient intensity. Conversely, sessions of moderate duration can produce robust gains when repetition density and engagement are sufficiently high.

Second, the model highlights that intensity is modifiable without additional resources. Reorganizing session structure, reducing inactive time, prioritizing task-specific practice, and actively engaging patients can dramatically increase effective dose without extending session length.

Third, this operationalization allows intensity to be monitored, adjusted, and individualized, paving the way for personalized rehabilitation rather than standardized time-based prescriptions. Importantly, it also addresses the therapeutic pessimism documented across Central African healthcare systems, where low-intensity conventional schedules are systematically prescribed based on prognosis rather than evidence (29). Replacing duration conventions with dose-informed prescription represents both a scientific and an ethical imperative in these contexts.

## From evidence to prescription

### Defining actionable intensity thresholds

While Tables 1, 2 establishes the superiority of intensity-centered paradigms across heterogeneous designs and contexts, clinical implementation requires a further step: defining actionable intensity targets. Without operational thresholds, intensity risks remaining a conceptual ideal rather than a prescribable parameter.

Drawing on the converging evidence synthesized above and on integrative clinical experience across high- and low-resource settings (8–11), phase-specific intensity zones are proposed to guide clinical decision-making. These thresholds reflect the interaction between neuroplastic responsiveness, patient tolerance, and safety constraints. Their derivation integrates the experience-dependent plasticity principles established by Kleim and Jones (33), the dose-response evidence of Kwakkel et al. (31) and Lohse et al. (32), and the phase-specific biological vulnerability data informing the acute phase thresholds (7). They should be interpreted as evidence-informed starting ranges requiring prospective validation, not as validated clinical standards, Table 3.

**Table 3:** Phase-adapted intensity zones for post-stroke neurorehabilitation.

Stroke phase	*I*_min (minimal effective intensity)	Target intensity zone	Upper caution threshold	Clinical rationale
Acute stabilized (Day 3–14)	I ≥ 10	*I* = 15–25	I ≥ 40	Initiate neuroplastic activation while preserving cardiovascular and neurological safety. Supported by early HIIT and monitored acute-phase trials (7). Peri-infarct tissue biologically vulnerable; intensity must be carefully titrated.
Early subacute (Week 2–6)	I ≥ 20	*I* = 30–50	I ≥ 60	Peak neuroplastic responsiveness. High-density, task-oriented and compressed protocols yield maximal gains (1, 3–6, 8). Kleim & Jones principles of specificity and repetition apply maximally (33).
Late subacute (Week 6–12)	I ≥ 20	*I* = 35–55	I ≥ 65	Consolidation and expansion of functional gains. Maintain intensity while preventing fatigue-related disengagement (4–6, 10). Dose-response curves begin to plateau; engagement component (E) becomes critical.
Chronic phase (over 6 months)	I ≥ 15	*I* = 30–45	I ≥ 55–60	Overcome learned non-use and therapeutic pessimism (10, 11, 29). Evidence supports efficacy even years post-stroke when thresholds are exceeded (1, 8–10). Therapeutic nihilism is the primary modifiable barrier in this phase.

Thresholds are derived from experience-dependent plasticity principles (33), meta-analytic dose-response data (31, 32), and integrative clinical evidence (1, 7–11). I values are computed using the formula *I* = (*R* × *L* × *E*)/*T*. These zones represent evidence-informed clinical guidance requiring prospective validation; they should not be applied as prescriptive standards without individual clinical assessment. I_min: minimal effective intensity; CIMT: constraint-induced movement therapy.

### Rationale for phase-specific thresholds

Neuroplastic potential is not static across the stroke continuum. Experimental and clinical data consistently indicate that responsiveness to training intensity varies across acute, subacute, and chronic phases, with distinct risks associated with both under-dosing and over-dosing at each stage.

In the acute stabilized phase, intensity must remain sufficient to stimulate neural activation while respecting cardiovascular and neurological safety constraints. Evidence from HIIT-based and early mobilization trials demonstrates that intensity can be safely initiated when progression is monitored and duration is controlled (7).

During the subacute phase, neuroplastic sensitivity reaches a peak. This window represents the most critical opportunity to deliver high-density, task-oriented practice. The superiority of compressed CIMT, high-repetition training, and intensive multimodal programs during this phase strongly supports aggressive—but structured—intensity escalation (1, 3–6, 8). The principles of experience-dependent plasticity established by Kleim and Jones provide the mechanistic framework underpinning these thresholds: specificity, repetition, intensity, and salience each independently contribute to cortical reorganization, and their integration within a structured dose framework is the translational contribution of the present model (33).

In the chronic phase, intensity remains effective but must be strategically targeted to overcome learned non-use and therapeutic pessimism. Evidence from Queen Square-type programs and Franco–Cameroonian intensive models demonstrates that meaningful recovery remains achievable years after stroke when repetition density and engagement thresholds are exceeded (1, 8–10). The pervasive therapeutic nihilism documented among healthcare professionals in Central Africa—where medical students demonstrated rehabilitation negativity indices significantly higher than stroke patients' families—underscores that the principal barrier to chronic-phase recovery is attitudinal rather than biological (29).

**Figure 1:**
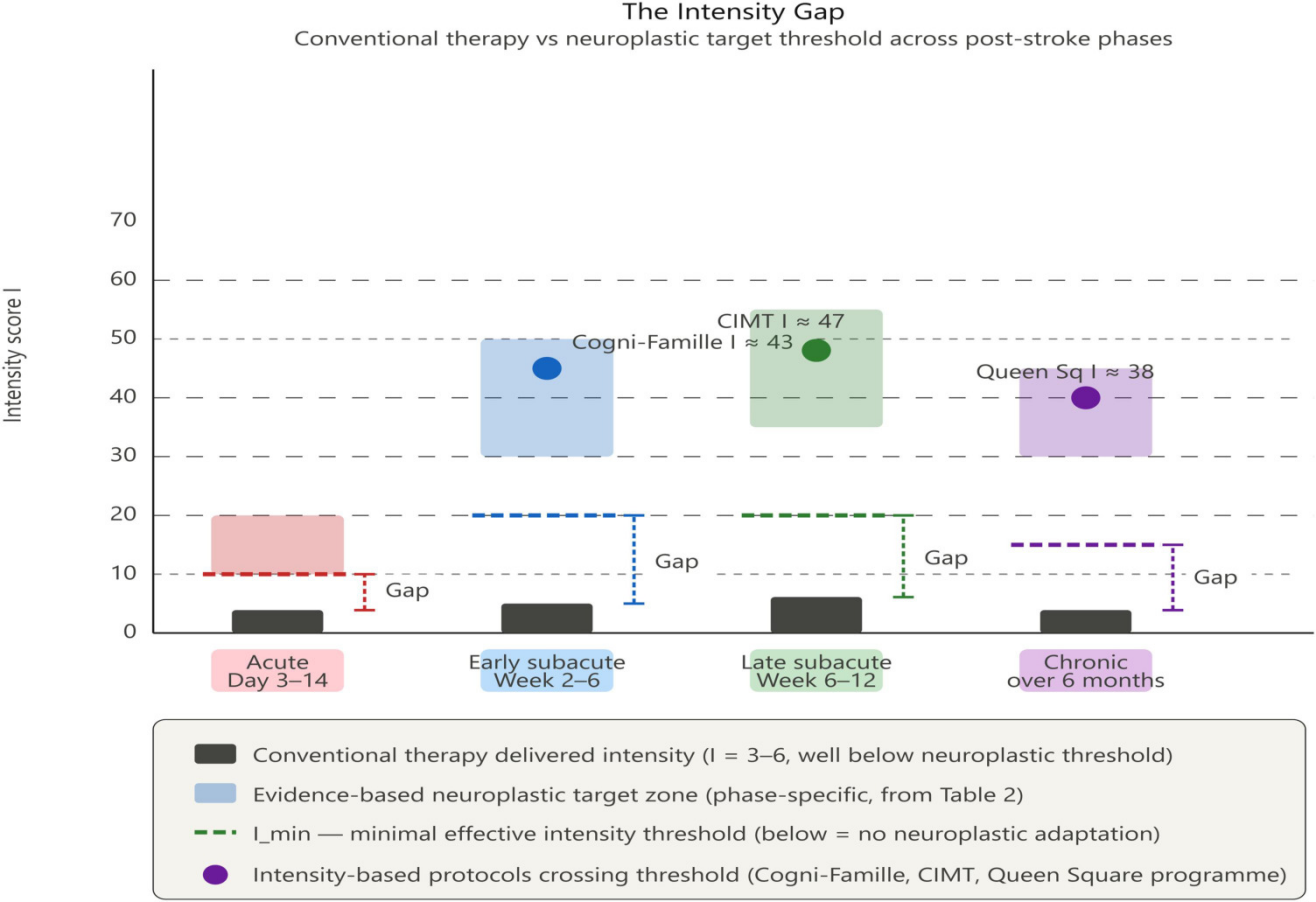
The intensity Gap: conventional therapy vs. neuroplastic target threshold across post-stroke phases. For each phase, the dark gray bar represents the typical intensity score delivered by conventional duration-based therapy (*I* = 3–6). The colored zone represents the evidence-based neuroplastic target range derived from Table 2. The dashed line marks the minimal effective intensity (*I*_min) below which neuroplastic adaptation is unlikely to occur. Colored dots indicate intensity scores achieved by evidence-based protocols that cross the threshold: Cogni-Famille family-mediated protocol (*I* ≈ 43) (9); compressed Constraint-Induced Movement Therapy (*I* ≈ 47) (4); Queen Square intensive upper limb programme (*I* ≈ 38) (1). Intensity scores are computed using the formula *I* = (*R* × *L* × *E*)/*T*, where *R* = task-oriented repetitions, *L* = neurophysiological load (composite score 1–12), *E* = proportion of repetitions above adaptive engagement threshold (0–1), and *T* = effective session time in minutes. Values are illustrative estimates derived from published protocol parameters; prospective validation of the scoring system is warranted. CIMT, constraint-induced movement therapy; *I*_min, minimal effective intensity.

 Figure 1 illustrates the core argument of this Perspective in a single visual: across all four post-stroke phases, conventional duration-based therapy consistently delivers intensity scores of 3–6, well below the minimal effective threshold (*I*_min) defined in Table 2. This “Intensity Gap” is not a resource problem—it is a prescription problem. Intensity-based protocols that explicitly target neuroplastic thresholds—the Cogni-Famille family-mediated model (*I* ≈ 43), compressed CIMT (*I* ≈ 47), and the Queen Square intensive programme (*I* ≈ 38)—cross these thresholds and account for the superior functional outcomes documented in Table 1. Closing this gap does not require additional technology or time; it requires a deliberate shift from duration-centered to dose-centered clinical reasoning.

### Avoiding two symmetrical errors: under-dosing and over-dosing

The integration of Tables 1, 3 highlights two frequent but symmetrical clinical errors. First, under-dosing through duration-centered prescriptions. Long sessions with low repetition density and passive components frequently fail to reach the minimal intensity threshold required to activate neuroplastic cascades, particularly in chronic stroke. Second, over-dosing without structure. Excessive intensity delivered without progression control or recovery planning may result in fatigue, disengagement, or adverse events, ultimately reducing therapeutic adherence. The solution lies not in maximal intensity, but in optimal intensity, dynamically adjusted across phases and patient profiles, Table 4.

**Table 4:** The intensity-dose conceptual model across the post-stroke continuum.

Acute Day 3–14	Early subacute Week 2–6	Late subacute week 6–12	Chronic over 6 months
*I*_min >=10 Target zone: 15–25 Caution: >=40	*I*_min >=20 Target zone: 30–50 Caution: >=60	*I*_min >=20 Target zone: 35–55 Caution: >= 65	*I*_min >=15 Target zone: 30–45 Caution: >=55–60
Biological vulnerability Careful titration required *HIIT safe if monitored* (7)	Peak plasticity window Max density protocols *CIMT* + *high-rep* (1, 3–6, 8)	Consolidation phase Fatigue monitoring critical *Engagement (E) dominant* (4–6, 10)	Recovery still possible Counter therapeutic nihilism (29) *Family-mediated models* (9)
*I* = (R x L x E)/T	Duration alone is INSUFFICIENT in all phases	Technology is NOT required	Family-mediated models match high-tech outcomes

The Intensity-Dose conceptual model across the post-stroke continuum. Each phase is characterized by its minimal effective intensity (I_min), target zone, and upper caution threshold, derived from experience-dependent plasticity principles (33), dose-response meta-analyses (31, 32), and phase-specific clinical evidence (1, 4, 7–11). The central message is constant across phases: duration alone is an insufficient prescription unit. *I* = (*R* × *L* *×* *E*)/*T*; see text for variable definitions. CIMT, constraint-induced movement therapy; I_min, minimal effective intensity.

*I* = (*R* × *L* × *E*)/*T*|Duration-based prescription FAILS in all phases if repetition density and engagement remain below *I*_min|Technology is NOT required: family-mediated and low-resource protocols can match high-tech outcomes when intensity is preserved.

## The 3P implementation framework: personalize-progress-prevent

To translate intensity science into safe and effective clinical practice, intensity must be implemented within a structured framework. Drawing on the evidence summarized in Table 1 and integrative conceptual work (10, 11), we propose the 3P Framework.

### Personalize

Intensity is not universal. Each patient has an individual intensity window defined by stroke severity, phase of recovery, comorbidities, fatigue profile, and cognitive capacity. The clinical challenge is therefore not whether to apply intensity, but how much, when, and for whom. Personalizing intensity thresholds is essential to avoid both under-dosing and overloading. This is particularly critical in resource-limited African settings where therapeutic nihilism systematically suppresses prescribed intensity below effective thresholds—a modifiable professional behavior, not an immutable constraint of the care environment (29).

### Progress

Neuroplastic responsiveness evolves over time. Evidence from acute, subacute, and chronic phases indicates that intensity should be progressively increased to match evolving adaptive capacity. Early stabilization phases require cautious initiation, while subacute windows allow rapid escalation. Importantly, chronic stroke does not preclude high-intensity gains when progression is structured and engagement is preserved.

### Prevent

Finally, intensity must be delivered safely. Excessive neurophysiological load without adequate recovery may lead to fatigue, disengagement, or adverse events. Monitoring physiological and cognitive fatigue, structuring recovery periods, and alternating high- and moderate-intensity days are essential to maintain long-term effectiveness.

## Clinical and system_level implication

### Rethinking rehabilitation dose beyond time-based metrics

The integration of Tables 1, 2 highlights a fundamental mismatch between contemporary neuroplasticity science and prevailing rehabilitation delivery models. While evidence consistently demonstrates that therapeutic intensity is the principal determinant of functional recovery, most rehabilitation systems continue to organize care around session duration and calendar time rather than biological dose.

### Implications for clinical practice

For clinicians, the shift from duration-centered to intensity-centered prescription requires a change in clinical reasoning rather than additional resources. As demonstrated across the paradigms summarized in Table 1, comparable intensity levels can be achieved through diverse means: repetition density, cardiovascular load, task salience, or caregiver-mediated amplification. The clinician's role therefore evolves from time allocation to dose optimization.

Table 2 provides a pragmatic scaffold to support this transition. By defining phase-adapted intensity zones, it allows clinicians to calibrate rehabilitation dose according to neuroplastic responsiveness, patient tolerance, and safety constraints. Importantly, this approach legitimizes high-intensity rehabilitation in chronic stroke when delivered within structured thresholds, countering persistent therapeutic nihilism—a clinical and educational problem of the first order in Central Africa, where healthcare professionals' negative prognosis expectations constitute an independent predictor of functional outcomes (29).

### Implications for training and professional culture

The persistence of duration-based prescriptions is partly rooted in professional training. Rehabilitation curricula frequently emphasize techniques and session formats while underemphasizing dose reasoning. Integrating intensity as a core pedagogical concept—alongside motor learning and neuroplasticity—would better equip future practitioners to design biologically effective interventions (26–28).

Family-mediated and low-resource models further challenge traditional professional boundaries. As shown in Table 1, intensity can be multiplied without proportional increases in professional time by redistributing selected tasks to trained caregivers. This does not dilute professional expertise; rather, it repositions the therapist as an architect of intensity, supervising and calibrating distributed therapeutic inputs. The Cogni-Famille model is the clearest existing proof of concept for this approach (9). Its transposability to musculoskeletal management has been documented in osteopathic practice, where a structured spine-centred protocol exceeded the minimally clinically important difference fourfold in a resource-limited African context (30).

### Implications for health systems and policy

At the system level, time-based reimbursement models risk structurally incentivizing low-intensity care. When funding is tied to session duration rather than delivered dose, there is little institutional motivation to optimize repetition density or engagement. This misalignment may partially explain why prolonged rehabilitation pathways often fail to translate into proportional functional gains.

Adopting intensity-centered metrics could inform more meaningful quality indicators, such as effective repetitions per session or engagement-adjusted therapy time. Such metrics would align resource allocation with biological effectiveness rather than administrative convenience. The WHO Africa Rehabilitation Strategy 2025–2035 provides a policy framework within which intensity-centered metrics could be embedded; without such integration, the risk of perpetuating under-dosing at scale remains high (26–28).

## Limitations and perspective scope

### Limitations and safety considerations

While this Perspective advocates for an intensity-centered approach to post-stroke neurorehabilitation, it is essential to emphasize that intensity is not synonymous with indiscriminate escalation. Neuroplastic responsiveness is dynamically modulated by the biological state of the injured brain, particularly during the early inflammatory and peri-lesional phases.

Experimental and clinical observations suggest that, in the acute and early subacute phases, the ischemic and peri-infarct tissue remains biologically vulnerable. During this window, excessive neurophysiological load—especially when combined with insufficient monitoring—may risk destabilizing homeostatic processes rather than promoting adaptive plasticity. This underscores the necessity of phase-specific intensity modulation, as reflected in the lower initiation thresholds and narrower target zones proposed in Table 2.

The quantification framework proposed here—*I* = (*R* × *L* × *E*)/*T*—is not a validated clinical scoring instrument. The equation functions as a conceptual scaffold for structured clinical reasoning, not as a precision tool. The components *L* (neurophysiological load) and *E* (engagement proportion) require prospective operationalization and inter-rater reliability testing before deployment as formal metrics. The intensity values proposed in Table 2 are derived from convergent evidence and clinical experience; they require prospective validation in controlled trials before adoption as clinical standards. This is a recognized and important limitation of the present work, consistent with the Perspective article format.

A second important limitation relates to patient-specific contraindications and systemic factors. High-intensity rehabilitation may be inappropriate or require substantial adaptation in the presence of uncontrolled cardiovascular instability, severe autonomic dysfunction, acute medical complications, or profound cognitive and attentional impairment. Furthermore, fatigue, pain, sleep deprivation, and affective disturbances can substantially reduce the effective engagement component of intensity, rendering escalation counterproductive.

These considerations reinforce a central message: intensity must be individualized, monitored, and progressively adjusted, not uniformly applied. The proposed intensity framework does not replace clinical judgment; it provides a structured lens through which clinicians can balance adaptive stimulation against biological vulnerability.

### Perspective

Beyond the immediate clinical implications, the intensity-centered framework proposed here opens several avenues for future development. A priority direction concerns the refinement of clinically feasible intensity metrics capable of capturing effective dose without excessive technological burden.

A second perspective relates to the integration of intensity-centered reasoning into education and professional culture. Current rehabilitation training programs remain largely technique-oriented, with limited emphasis on dose calibration and neuroplastic thresholds. Embedding intensity as a core conceptual pillar would better prepare clinicians to design biologically effective interventions across diverse contexts—an imperative underscored by the structural barriers to rehabilitation education in Central Africa identified across multiple studies (26–28). An AI-assisted functional scoring system (Projet REHAB-AI, Université de Dschang–Sorbonne Université partnership) currently under development could offer a scalable low-cost solution for intensity monitoring in resource-limited settings.

Finally, the framework proposed here invites a rethinking of health system organization and research priorities. Future clinical trials should prioritize intensity-informed outcomes, exploring how adaptive dose modulation influences long-term recovery, sustainability, and equity.

## Conclusion

Across contemporary post-stroke neurorehabilitation paradigms, a consistent and compelling message emerges: functional recovery is not primarily determined by how long therapy lasts, but by how much biologically effective stimulation is delivered per unit time. Duration-centered prescriptions, although administratively convenient, remain poor surrogates for therapeutic dose and risk perpetuating systematic under-dosing across all phases of stroke recovery.

The evidence synthesized in Table 1 demonstrates that when repetition density, neurophysiological load, and patient engagement are optimized, meaningful recovery can be achieved across diverse clinical contexts, independently of technological sophistication or resource availability. By operationalizing therapeutic intensity and translating it into phase-adapted intensity zones (Table 2) and a conceptual dose model (Figure 1), this Perspective moves beyond conceptual advocacy toward actionable clinical guidance.

Importantly, this framework addresses two converging barriers to effective post-stroke neurorehabilitation: the structural barrier of duration-based prescription systems, and the attitudinal barrier of therapeutic nihilism—documented to be paradoxically amplified by medical training in Central African healthcare contexts (29). Both barriers are modifiable, and both can be addressed through the same conceptual shift: from time to dose, from nihilism to precision.

Prescribing therapy in biologically meaningful units of intensity—personalized, progressive, and safely constrained—offers a unifying framework capable of bridging technological, cultural, and resource divides. In this sense, intensity is not an adjunct to rehabilitation dose; it is its core determinant.

## Clinical note

From a practical standpoint, the intensity-centered framework proposed in this Perspective invites clinicians to reconsider how rehabilitation sessions are structured on a day-to-day basis. Rather than extending session duration, immediate gains can often be achieved by reorganizing existing time to minimize inactive periods, prioritize task-specific practice, and actively monitor patient engagement. Simple adjustments—such as reducing passive transitions, increasing meaningful repetitions, or clustering tasks within focused time windows—may substantially increase effective therapeutic dose without additional resources.

Clinically, intensity should always be interpreted through the lens of biological readiness. In early post-stroke phases, particularly during periods of heightened neuroinflammation, clinicians should favor carefully titrated intensity with close monitoring of fatigue, autonomic responses, and cognitive load. In contrast, during subacute and chronic phases, insufficient intensity remains a more frequent limitation than excessive load.

This framework reinforces the role of the rehabilitation professional as a dose strategist rather than a time provider. Whether working in high-technology environments or low-resource settings, clinicians retain full agency over intensity modulation through clinical reasoning, task selection, and engagement facilitation. When intensity is prescribed deliberately—personalized, progressive, and safely constrained—it becomes a powerful lever to overcome therapeutic stagnation and restore meaningful functional recovery across the post-stroke continuum.

In short, **intensity is not an adjunct to rehabilitation dose; it is its core determinant**.

## Data Availability

The original contributions presented in the study are included in the article/Supplementary Material, further inquiries can be directed to the corresponding author.
